# Distribution of alpine endemic plants of northern Asia: a dataset

**DOI:** 10.3897/BDJ.9.e75348

**Published:** 2021-10-14

**Authors:** Elena Brianskaia, Denis Sandanov, Yichao Li, Zhiheng Wang

**Affiliations:** 1 Institute of General and Experimental Biology, Ulan-Ude, Russia Institute of General and Experimental Biology Ulan-Ude Russia; 2 Peking University, Beijing, China Peking University Beijing China

**Keywords:** dataset, endemic alpine plants, northern Asia, digitising printed maps

## Abstract

**Background:**

We describe a dataset providing information on the geographic distribution of northern Asian endemic alpine plants. It was obtained by digitising maps from the atlas “Endemic alpine plants of Northern Asia”. Northern Asia includes numerous mountain ranges which may have served as refugia during the Pleistocene ice ages, but there have been no studies that analysed this question. We suggest that this dataset can be applied for better understanding of the alpine endemism in northern Asia.

**New information:**

The dataset includes 13709 species distribution records, representing 211 species from 31 families and 106 genera. Each record provides data regarding the distribution of an individual species. These data provide a foundation for studying northern Asia's endemic alpine species and conducting research on the factors concerning their distribution.

## Introduction

Being climatically and topographically heterogeneous, mountain ecosystems are characterised by a high degree of plant species diversity (López-Pujol et al. 2011, Hassan et al. 2005). They are often considered to have been potential refugia or buffering zones that either prevented extinction or promoted speciation during the Quaternary glacial-interglacial shifts because of their high spatiotemporal climatic stability (Sandanov et al. 2020, Harrison and Noss 2017, Feng et al. 2016, Sandel et al. 2011). During the Pleistocene glacial periods, ice sheets expanded greatly throughout northern Asia, mountainous regions contributed to the preservation of a number of alpine species (Harrison and Noss 2017, Volkova and Baranova 1980). Malyshev considered nine mountain areas in northern Asia that served as refugia during ice ages of Pleistocene for at least 231 alpine endemic species (Malyshev 1979, Vodopyanova et al. 1974). Alpine endemism was studied for mountain ranges of Siberia, Far East and northern part of Asia (Malyshev 1972, Krasnoborov 1974, Yurtzev 1981, Schlothauer 1990). In more recent studies, it was revealed that Far East has seven centres of edemism (Kozhevnikov 2007). However, despite numerous studies on endemism of northern Asia's alpine plants, it is not considered as an endemism hot spot on a global scale (Harrison and Noss 2017, Hobohm et al. 2019). Moreover, to this date, there have been no studies that quantitatively assess the correlation amongst climate, topography and alpine endemism in northern Asia. We consider the lack of baseline species distribution data is the main reason for this lack. We have developed and are sharing this dataset to address this need and to encourage the quantitative analyses required for developing a better understanding of the alpine endemism in northern Asia (Brianskaia et al. 2021).

## General description

### Purpose

Our primary goal was to digitise species distribution maps of alpine endemics of northern Asia and to encourage use of the data developed.

### Additional information

**Figure 1.**
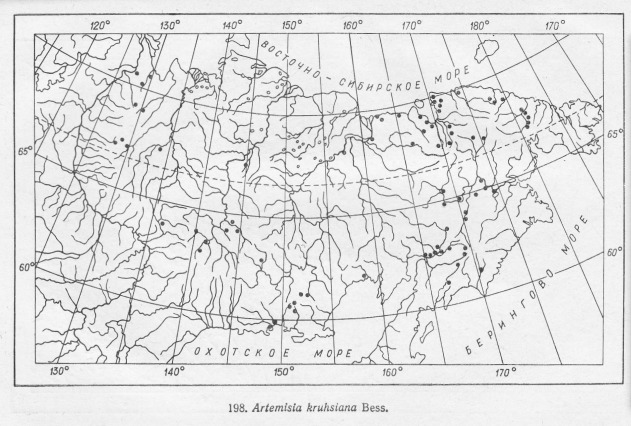
Example of the distribution map scan – *Artemisiakruhsiana*.

**Figure 2.**
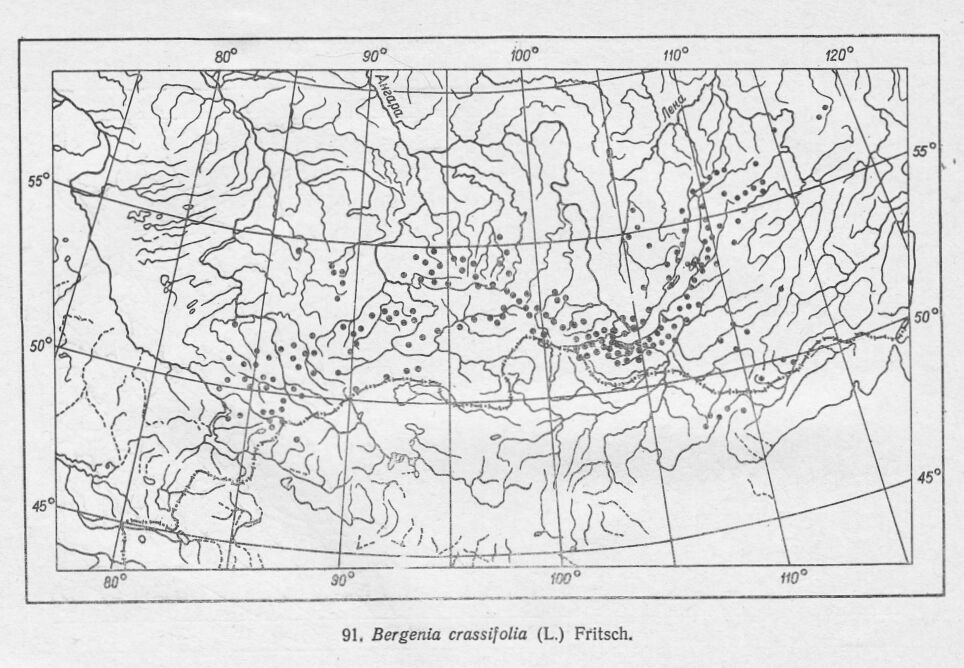
Example of the distribution map scan – *Bergeniacrassifolia*.

Studies of the geographic distribution of endemic alpine plants were very significant in the Soviet botany of the 60s-70s (Tolmachev 1962, Malyshev 1965, Yurtzev 1966, Yurtzev 1968). In 1965, the Soviet commission of the flora and vegetation history sponsored a project on the sudy of northern Asia's endemic plants, particularly the endemic species. The printed atlas was the result of teamwork by the Siberian and All-Soviet Union botanists. The data for the atlas were compiled from the Leningrad (LE), Moscow (MW), Ural (SVER), Siberian (NSK, NS, TK, IRK, SASY) and Far East (VLA) herbaria. The atlas includes a list of the endemic alpine plant species of northern Asia with their habitat characteristics, geographic range and cartographic materials. The distribution maps (Figs 1, 2) were prepared from herbarium specimens whose identification had been checked (Vodopyanova et al. 1974). The list of editors of the atlas includes Vodopyanova N.S., Malyshev L.I., Siplivinskiy V.N., Tolmachev A.I. and Yurtsev B.A. Many different cartographers were involved in preparing the published maps (Table 1). Taxonomy of species in the GBIF dataset is given both as published in the atlas (Vodopyanova et al. 1974) in scientificName column and verified according to the Catalogue of Life (Roskov et al. 2019) and Checklist of Asian Russia Flora (Baikov 2012). The final verified taxonomy was checked with GBIF species matching tool and given in the acceptedNameUsage column.

## Project description

### Title

№ 121030900138-8 «Biota of terrestrial ecosystems of Baikal Region: composition, structure, eco-geographic patterns»

### Personnel

Elena Brianskaia, Denis Sandanov

### Study area description

Baikal Region, Russia

### Design description

The project carries out studies in different disciplines: flora and plant taxonomy, plant biology and population ecology, vegetation of Baikal Region, fauna and ecology of insects, ecology and geography of vertebrates.

### Funding

Russian Federal Budget

## Sampling methods

### Study extent

**Figure 3.**
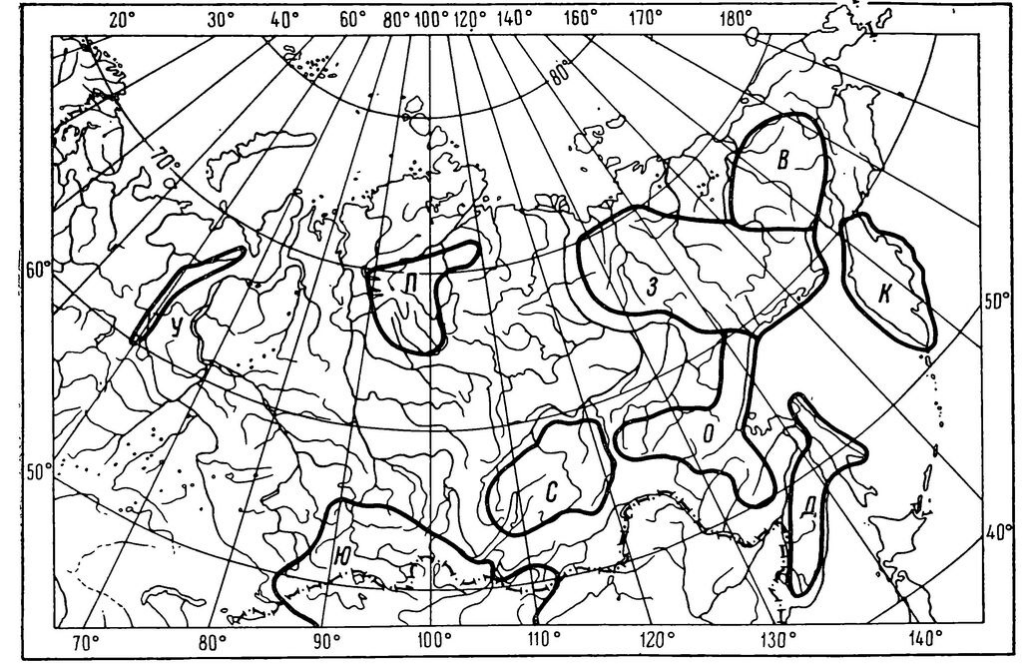
The territory of northern Asia and areas with alpine flora (Malyshev 1979): У – Urals; П – Putorana Plateau; З – the western part of Verkhoyano-Kolymskaya mountain range; В – the eastern part of Verkhoyano-Kolymskaya mountain range; К – Kamchatka; Ю – Southern Siberia and Mongolia; С – Stanovoy highlands; О – Priokhotskiy highlands: Stanovoy, Dzhugzhur, Ezop mountain ranges etc.; Д – the southern part of Russian Far East: Primor’e and Sakhalin.

Northern Asia is an extensive area, stretching from the Ural Mountains in the west to the Pacific Ocean in the east; from the Arctic Ocean in the north to Central and East Asia in the south. According to Malyshev (Malyshev 1979), there are nine areas with alpine flora which includes mountains of Russian Far East, south-eastern Siberia, Ural and Putorana (Fig. 3).

### Sampling description

In total, 231 maps were scanned from the atlas Endemic Alpine Plants of Northern Asia (Vodopyanova et al. 1974). All maps were adjusted to the same size and horizontal position in order to obtain standardised images of the maps. Digitalisation was performed in QGIS 3.10 software by means of a georeferencing tool. Source raster distribution maps were georeferenced by snapping control points to the destination vector shapefile, which, in our case, was the border of Russia. This transformed all the maps to the WGS84 spatial projection. Subsequently, species distribution locations were digitised from each map. Coordinates of each location were calculated by QGIS and displayed in the attribute table.

### Quality control

Final examination of the digitised species distribution maps was performed in QGIS 3.10. This step took most of the time and efforts in the entire digitising process. Each digitised map was compared to the original print map and the habitat of each digitised record compared with the habitat characteristics and geographic range of the species concerned reported in literature. Major sources for this part of the review were the Flora of Siberia (Krasnoborov et al. 1997, Lomonosova et al. 1992, Malyshev et al. 1990, Peshkova et al. 1994, Peshkova et al. 1990, Pimenov et al. 1996, Polozhiy et al. 1994, Polozhiy et al. 1996, Timokhina et al. 1993, Vlasova et al. 1987, Vydrina et al. 1998, Doronkin et al. 1997, Kashina et al. 1988) and Vascular Plants of Soviet Far East (Kharkevich 1985, Kharkevich 1987, Kharkevich 1988, Kharkevich 1989, Kharkevich 1991, Kharkevich 1992, Kharkevich 1995, Kharkevich 1996). Almost all (97%) of the digitised maps were consistent with the printed maps. Those that were not included records from near the ocean and in the Far East. They were manually adjusted to match the printed maps. For example, such records were adjusted for *Betulamiddendorffii* for which distribution goes along the sea of Okhotsk (Figs 5, 6, 7, 8). оклонения от речной сети размер точек

Coordinate uncertainty in metres was calculated, based on three types of uncertainties (Chapman and Wieczorek 2020). The first type is the coordinate uncertainty of the species occurrence from the herbarium locality description. As mentioned earlier, the maps in the atlas were drawn, based on the herbaria specimen. In order to test this type of coordinate uncertainty, the occurrence dataset from the Moscow University Herbarium (MW) was used as the reference (Seregin 2021). A total of 1500 random occurrences from the Asian part of Russia were taken from MW herabrium and analysed. Generally, the coordinate uncertainty for all analysed occurrences ranges from 0.1 to 60 km. All the data were divided in three random groups by 500 occurrences. The mean coordinate uncertainty for each group equals to 3.86, 5.66 and 2.96 km. Thus, the mean value amongst these three groups was close to 4 km. Based on this result, we established approximately 5 km as the coordinate uncertainty.

The second type is the coordinate uncertainty of the drawn maps. The endemic plants of the northern Asia atlas includes four types of maps: a) for the entire northern Asia; b) for northern Asia from 120^0^ to 170^0^ E; c) for south Siberia from 75^0^ to 120^0^ E; d) for Far East including Kamchatka Peninsula, Sakhalin Island and Kuril Islands. The coordinate uncertainty of distribution records on each type of the map varies due to its scale. The calculation of coordinate uncertainty of the drawn maps was performed by measuring the distance between species distribution records and the closest river drainage in QGIS 3.10. River drainage was crucial in Soviet botanical mapping as it was used as the reliable feature for species occurrence location. In order to calculate the average coordinate uncertainty, each distance was summarised and divided by the number of measurements. Thus, a) 30 km is the uncertainty for distribution records of the entire northern Asia maps; b) 25 km for the northern Asia maps from 120^0^ to 170^0^ E; c) 20 km for the south Siberia maps from 75^0^ to 120^0^ E; d) 15 km for the Far East maps including Kamchatka Peninsula, Sakhalin Island and Kuril Islands.

The third type is the coordinate uncertainty of the map digitalisation in QGIS 3.10. To test the coordinate uncertainty of the map digitalisation, three experts independently performed it on their computers for each of four types of the maps. As a result, the coordinate uncertainty was less than 5 km in all cases in all types of the maps by three experts.

The final coordinate uncertainty was calculated by summariing all three above-mentioned uncertainties for four types of maps.

## Geographic coverage

### Description

**Figure 4.**
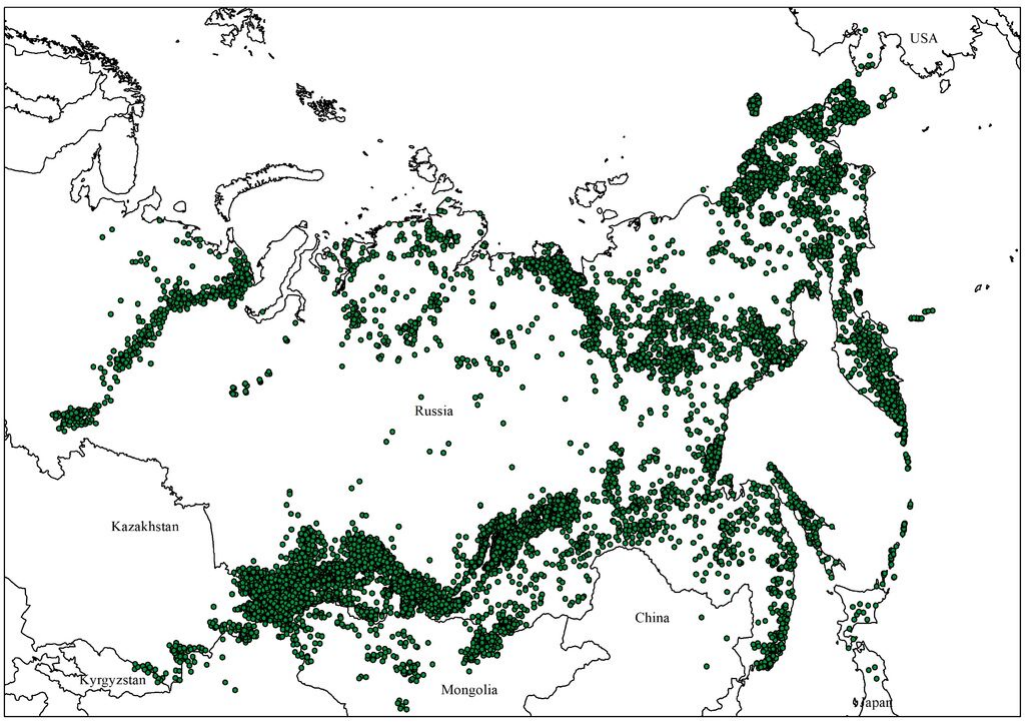
Geographic coverage of distribution records.

**Figure 5.**
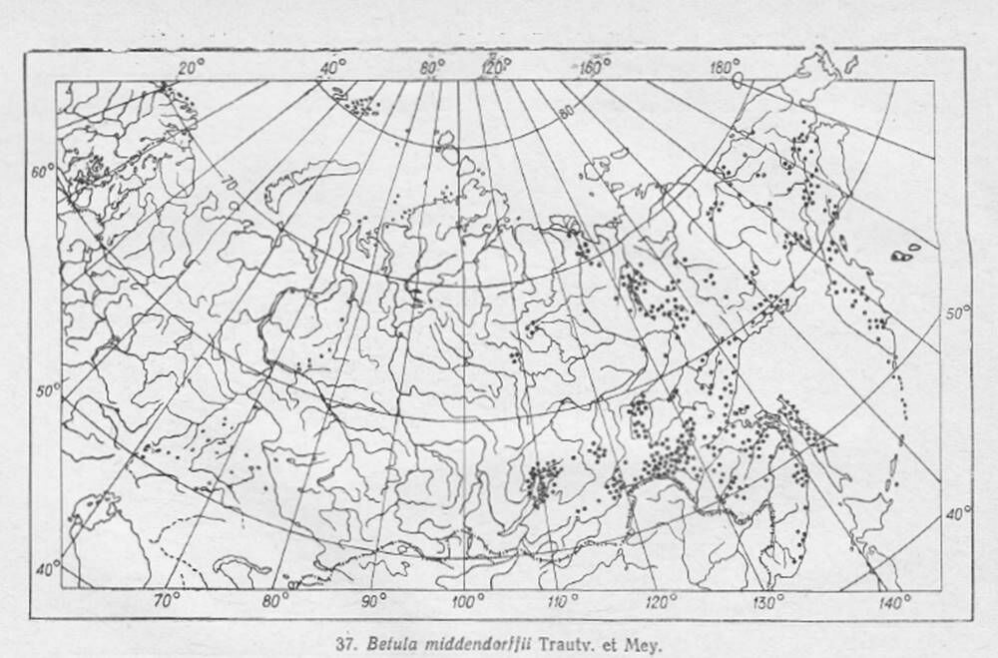
*Betulamiddendorffii* distribution map scan.

**Figure 6.**
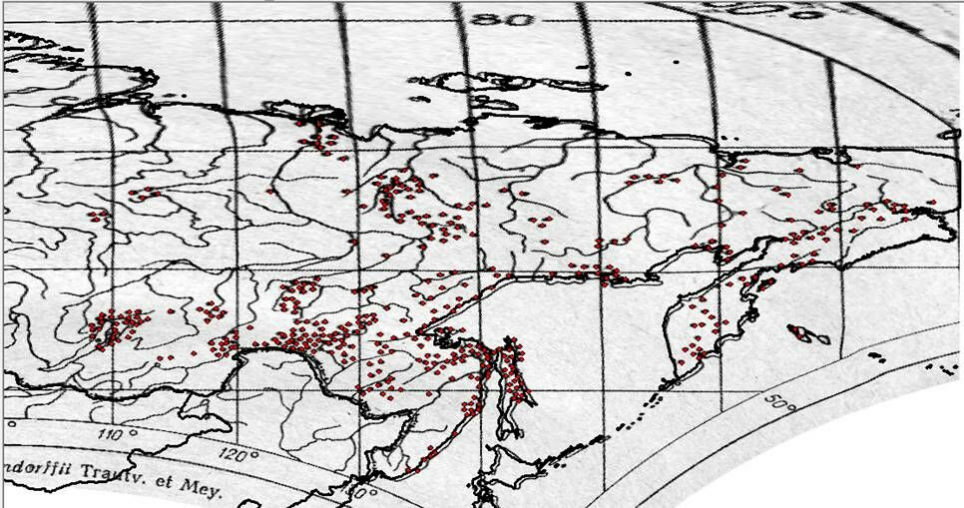
Georeferencing of *Betulamiddendorfii* distribution map in QGIS 3.10.

**Figure 7.**
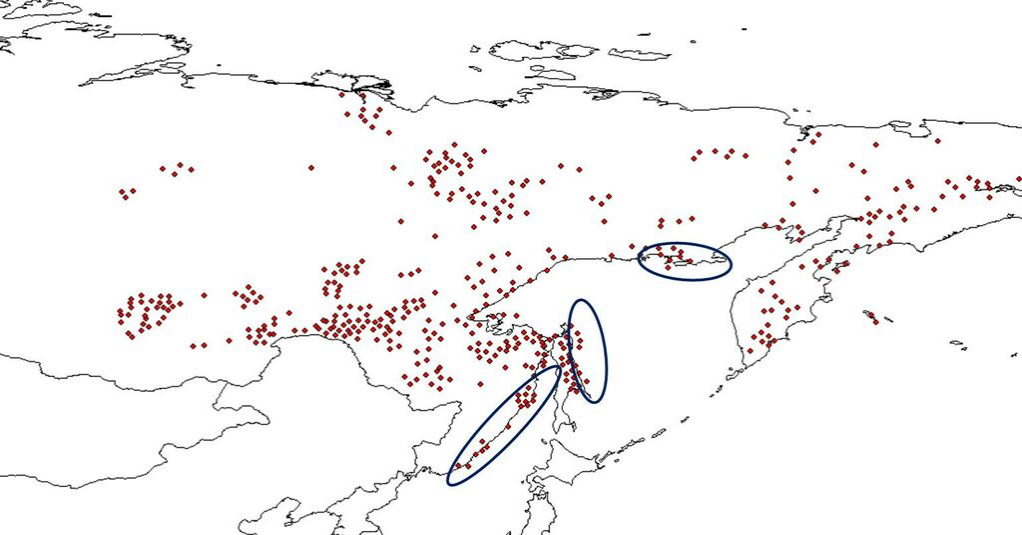
The example of *Betulamiddendorffii* distribution records being digitised out of the shapefile in QGIS 3.10.

**Figure 8.**
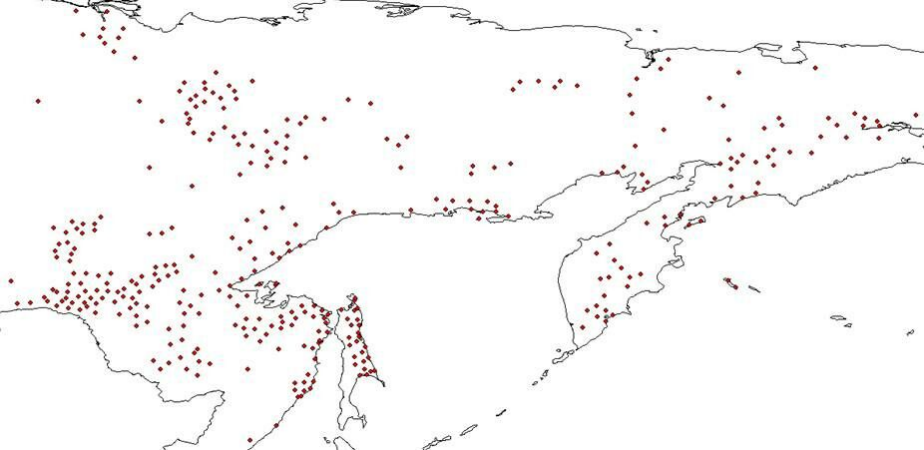
The result of *Betulamiddendorffii* distribution records adjusted by reference to their original location in QGIS 3.10.

The majority of the records were located within Russia (12762 records – 93.1%; Fig. 4), but a few came from other countries: Mongolia (440 records – 3.2%), Kazakhstan (414 records – 3%), China (58 records – 0.4%), Japan (16 records – 0.1%), USA, Alaska (12 records – 0.1%) and Kyrgyzstan (7 records – 0.05%).

### Coordinates

38.723405 and 77.563972 Latitude; 179.986717 and -179.985022 Longitude.

## Taxonomic coverage

### Description

In total, the dataset includes 231 species with 13709 distribution records from 31 families and 106 genera. The top 10 families hold 64% (8783 records) of the total number of endemic alpine species distribution records (Table 2). Additionally, a number of species distribution records were compiled for each species (Table 3). There are 44 species with distribution records > 10; 31 species > 20; 29 species >30; 32 species > 50; 46 species > 100; 49 species < 100.

## Temporal coverage

### Notes

Dates of records range from 1913 to 1972 and were published in 1974.

## Usage licence

### Usage licence

Creative Commons Public Domain Waiver (CC-Zero)

### IP rights notes

This work is licensed under a Creative Commons Attribution (CC-BY) 4.0 Licence.

## Data resources

### Data package title

Endemic Alpine Plants of Northern Asia

### Resource link

https://www.gbif.org/dataset/8ee10704-0472-4b5b-aa95-99748552c09c

### Alternative identifiers

https://doi.org/ 10.15468/96hq83

### Number of data sets

1

### Data set 1.

#### Data set name

Endemic Alpine Plants of Northern Asia

#### Data format

Darwin Core

#### Number of columns

29

#### Data format version

1.0

#### Description

We describe a dataset providing information on the geographic distribution of northern Asian endemic alpine plants. It was obtained by digitising maps from the atlas “Endemic alpine species of Northern Asia” (1974). The dataset includes 13709 species distribution records, representing 211 species from 31 families and 106 genera. Each record provides data regarding the distribution of an individual species.

**Data set 1.** 

Column label	Column description
eventDate	The date-time or interval during which an Event occurred. For occurrences, this is the date-time when the event was recorded. Not suitable for a time in a geological context. http://rs.tdwg.org/dwc/terms/eventDate
eventRemarks	Comments or notes about the Event. http://rs.tdwg.org/dwc/terms/eventRemarks
type	The nature or genre of the resource. http://purl.org/dc/elements/1.1/type
continent	The name of the continent in which the Location occurs. http://rs.tdwg.org/dwc/terms/continent
acceptedNameUsage	The full name, with authorship and date information if known, of the currently valid (zoological) or accepted (botanical) taxon in the online version. http://rs.tdwg.org/dwc/terms/acceptedNameUsage
scientificName	The full scientific name as in the "Endemic alpine plants of Northern Asia" 1974. http://rs.tdwg.org/dwc/terms/scientificName
occurrenceID	An identifier for the Occurrence (as opposed to a particular digital record of the occurrence). In the absence of a persistent global unique identifier, construct one from a combination of identifiers in the record that will most closely make the occurrenceID globally unique. http://rs.tdwg.org/dwc/terms/occurrenceID
kingdom	The full scientific name of the kingdom in which the taxon is classified. http://rs.tdwg.org/dwc/terms/kingdom
phylum	The full scientific name of the phylum or division in which the taxon is classified. http://rs.tdwg.org/dwc/terms/phylum
class	The full scientific name of the class in which the taxon is classified. http://rs.tdwg.org/dwc/terms/class
order	The full scientific name of the order in which the taxon is classified. http://rs.tdwg.org/dwc/terms/order
family	The full scientific name of the family in which the taxon is classified. http://rs.tdwg.org/dwc/terms/family
genus	The full scientific name of the genus in which the taxon is classified as in the "Endemic alpine plants of Northern Asia" 1974. http://rs.tdwg.org/dwc/terms/genus
specificEpithet	The name of the first or species epithet of the scientificName as in the "Endemic alpine plants of Northern Asia" 1974. http://rs.tdwg.org/dwc/terms/specificEpithet
taxonRank	The taxonomic rank of the most specific name in the scientificName. http://rs.tdwg.org/dwc/terms/taxonRank
decimalLatitude	The geographic latitude (in decimal degrees, using the spatial reference system given in geodeticDatum) of the geographic centre of a Location. http://rs.tdwg.org/dwc/terms/decimalLatitude
decimalLongitude	The geographic longitude (in decimal degrees, using the spatial reference system given in geodeticDatum) of the geographic centre of a Location. http://rs.tdwg.org/dwc/terms/decimalLongitude
geodeticDatum	The ellipsoid, geodetic datum or spatial reference system (SRS) upon which the geographic coordinates given in decimalLatitude and decimalLongitude are based. http://rs.tdwg.org/dwc/terms/geodeticDatum
coordinateUncertaintyInMetres	The horizontal distance (in metres) from the given decimalLatitude and decimalLongitude describing the smallest circle containing the whole of the Location.http://rs.tdwg.org/dwc/terms/coordinateUncertaintyInMeters
verbatimCoordinateSystem	The coordinate format for the verbatimLatitude and verbatimLongitude or the verbatimCoordinates of the Location. http://rs.tdwg.org/dwc/terms/verbatimCoordinateSystem
georeferencedBy	A list (concatenated and separated) of names of people, groups or organisations who determined the georeference (spatial representation) for the Location. http://rs.tdwg.org/dwc/terms/georeferencedBy
higherGeography	A list (concatenated and separated) of geographic names less specific than the information captured in the locality term. http://rs.tdwg.org/dwc/terms/higherGeography
country	The name of the country or major administrative unit in which the Location occurs. http://rs.tdwg.org/dwc/terms/country
countryCode	The standard code for the country in which the Location occurs. http://rs.tdwg.org/dwc/terms/countryCode
language	A language of the resource. http://purl.org/dc/elements/1.1/language
licence	A legal document giving official permission to do something with the resource. http://purl.org/dc/terms/license
associatedReferences	A list (concatenated and separated) of identifiers (publication, bibliographic reference, global unique identifier, URI) of literature associated with the Occurrence. http://rs.tdwg.org/dwc/terms/associatedReferences
basisOfREcords	The specific nature of the data record. http://rs.tdwg.org/dwc/terms/basisOfRecord
infraspecificEpithet	The name of the lowest or terminal infraspecific epithet of the scientificName as in the "Endemic alpine plants of Northern Asia" 1974. http://rs.tdwg.org/dwc/terms/infraspecificEpithet

## Figures and Tables

**Table 1.** List of cartographers of each species distribution map. *in acceptedNameUsage, column numbers are given for the type of the map the species are drawn in: 1) the entire northern Asia maps; 2) the northern Asia maps from 120^0^ to 170^0^ E; 3) South Siberia maps from 75^0^ to 120^0^ E; d) Far East maps including Kamchatka Peninsula, Sakhalin Island and Kuril Islands.

scientificName	acceptedNameUsage*	Cartographer
*Cryptogrammaraddeana*, *Dracocephalumfragile*, *Rhaponticumcarthamoides*, *Crepispolytricha*	*Cryptogrammaraddeana* (1), *Dracocephalumfragile* (3), *Forniciumcarthamoides* (3), *Crepispolytricha* (3)	Busik V.V.
*Microbiotadecussata*	*Microbiotadecussata* (4)	Gurzenkov N.N., Gorovoy P.G.
*Juniperuspseudosabina*	*Juniperuspseudosabina* (3)	Krasnoborov I.M. in consultation with Bardunov L.V., Goloskokov V.P., Kamelin R.V., Kashina L.I., Matsenko A.V.
*Ptilagrostisjunatovii*, *Koeleriageniculata*, *Poaivanoviae*, *Festucasichotensis*, *Roegneriasajanensis*, *Delphinumsajanense*, *Eutremaparviflorum*, *Drabapygmaea*, *Rhodiolapinnatifida*, *Saxifragabrachypetala*, *Chrysospleniumalbertii*, *Ch.peltatum*, *Oxytropisjurtzevii*, *O.sajanensis*, *Pinguiculaalgida*, *P.spathulata*, *Pyrethrumlanuginosum*, *Saussureasquarrosa*	*Ptilagrostisjunatovii* (3), *Koeleriageniculata* (3), *Poaivanoviae* (3), *Festucasichotensis* (1), *Elymussajanensis* (3), *Delphinumsajanense* (3), *Eutremaedwardsii* (3), *Drabapygmaea* (3), *Rhodiolapinnatifida* (3), *Saxifragabrachypetala* (3), *Chrysospleniumalbertii* (3), *Ch.peltatum* (3), *Oxytropisjurtzevii* (3), *O.sajanensis* (3), *Pinguiculaalgida* (1), *P.spathulata* (1), *Pyrethrumlanuginosum* (3), *Saussureasquarrosa* (3)	Malyshev L.I.
*Helictotrichonkrylovii*, *Poalanatiflora*	*Helictotrichonkrylovii* (2), *Hyalopoalanatiflora* (2)	Yurtsev B.A. in consultation with Mikhalyova V.M.
*Helictotrichonmongolicum*	*Helictotrichonmongolicum* (3)	Malyshev L.I. in consultation with Vodopyanova N.S.
*Koeleriaatroviolacea*	*Koeleriaatroviolacea* (3)	Gudoshnikov S.V.
*Poaaltaica*	*Poaaltaica* (3)	Vodopyanova N.S., Gudoshnikov S.V., Penkovskaya E.F. in consultation with Busik V.V., Goloskokov V.P., Ivanova M.M., Malyshev L.I.
*Poaircutica*, *Salixnasarovii*, *S.torulosa*, *Chrysospleniumbaicalense*, *Oxytropiskusnetzovii*, *O.oxyphylloides*, *Swertiabaicalensis*	*Poaircutica* (3) , *Salixnasarovii* (3), *S.torulosa* (3), *Chrysospleniumbaicalense* (3), *Oxytropiskusnetzovii* (3), *O.oxyphylloides* (3), *Swertiabaicalensis* (3)	Ivanova M.M.
*Poapseudobbreviata*	*Poapseudobbreviata* (1)	Matveeva N.V. in consultation with Yurtsev B.A., Malyshev L.I.
*Colpodiumaltaicum*	*Paracolpodiumaltaicum* (3)	Bardunov L.V. in consultation with Gudoshnikov S.V.
*Festucachionobia*	*Festucachionobia* (3)	Siplivinskiy V.N. in consultation with Busik V.V.
*Leymusinterior*	*Leymusinterior* (1)	Matveeva N.V.
*Eriophorumhumile*	*Eriophorumhumile* (1)	Petrovskiy V.V., Taraskina N.N. in consultation with Krasnoborov I.M. & Petrochenko Yu.N.
*Baethryonuniflorum*	*Kreczetovicziauniflora* (1)	Petrochenko Yu.N.
*Scirpusmaximowiczii*	*Scirpusmaximowiczii* (1)	Taraskina N.N. in consultation with Alyanskaya N.S., Bogdanova T.V., Maximova M.M., Malyshev L.I. & Yurtsev B.A.
*Carexalticola*, *Saxifragakruhsiana*	*Carexalticola* (1) , *Saxifragakruhsiana* (4)	Siplivinskiy V.N.
*Carexkaracolica*, *Oxytropissumneviczii*	*Carexcaucasica* (3), *Oxytropissumneviczii* (3)	Polozhiy A.V.
*Carexledebouriana*	*Carexledebouriana* (1)	Siplivinskiy V.N. in consultation with Busik V.V., Kashina L.I., Mikhalyova V.M., Penkovskaya E.F.
Luzulaunalaschkensisssp.kamtschadalorum	*Luzulaarcuata* (1)	Ivanova M.M. in consultation with Siplivinskiy V.N.
Salixberberifoliassp.berberifolia, S.berberifoliassp.brayi, S.berberifoliassp.fimbriata, S.berberifoliassp.kamtschatica	Salixberberifoliassp.berberifolia (3), *S.myrsinites* (3), *S.fimbriata* (1), *S.kamtschatica* (4)	Ivanova M.M., Gudoshnikov S.V. in consultation with Malyshev L.I. & Vodopyanova N.S.
*Salixdivaricata*	*Salixdivaricata* (3)	Ivanova M.M. in consultation with Vodopyanova N.S., Derviz-Sokolova T.G. & Malyshev L.I.
*Salixjurtzevii*, *S.khokhrjakovii*, *Cardamineconferta*, *C.pedata*, *C.victoris*, *Arabisturczaninovii*, *Saxigragaredowskii*, *Oxytropissemiglobosa*, *Androsacegorodkovii*, *A.semiperennis*, *Taraxacumsoczavai*	*Salixjurtzevii* (2), *S.khokhrjakovii* (2), *Corydalisgorodkovii* (2), *Cardamineconferta* (2), *C.pedata* (4), *C.victoris* (2), *Arabisturczaninovii* (2), *Saxifragaredofskyi* (2), Oxytropisajanensisssp.semiglobosa (2), *Androsacegorodkovii* (2), *A.semiperennis* (2), *Taraxacumsoczavae* (2)	Yurtsev B.A.
*Salixrectijulis*	*Salixrectijulis* (3)	Ivanova M.M., Gudoshnikov S.V. in consultation with Krasnoborov I.M. & Derviz-Sokolova T.G.
*Salixsajanensis*	*Salixsajanensis* (3)	Ivanova M.M. in consultation with Vodopyanova N.S. & Penkovskaya E.F.
*Salixsphenophylla*	*Salixsphenophylla* (1)	Petrovskiy V.V. & Taraskina N.N.
*Salixtschuktschorum*	*Salixtschuktschorum* (2)	Derviz-Sokolova T.G. in consultation with Taraskina N.N., Khokhryakov A.P. & Yurtsev B.A.
*Salixturczanonowii*	*Salixturczanonowii* (3)	Ivanova M.M., Krasnoborov I.M. in consultation with Malyshev L.I. & Siplivinskiy V.N.
*Betulamiddendorffii*	*Betulamiddendorffii* (1)	Ogureeva G.N.
*Betularotundifolia*	Betulananassp.rotundifolia (3)	Vodopyanova N.S., Krasnoborov I.M in consultation with Goloskokov V.P., Ivanova M.M., Kashina L.I. & Malyshev L.I.
*Claytoniaacutifolia*	*Claytoniaacutifolia* (1)	Matveeva N.V., Volkova E.V. in consultation with Yurtsev B.A. & Taraskina N.N.
*Claytoniaarctica*	*Claytoniaarctica* (2)	Matveeva N.V. in consultation with Volkova E.V. & Yurtsev B.A.
*Claytoniaeschscholtzii*	*Claytoniaeschscholtzii* (1)	Matveeva N.V., Volkova E.V. in consultation with Taraskina N.N. & Yurtsev B.A.
*Claytoniajoanneana*	*Claytoniajoanneana* (1)	Matveeva N.V., Volkova E.V. in consultation with Krasnoborov I.M. & Malyshev L.I.
*Claytoniellavassilievii*	*Claytoniavassilievii* (2)	Yurtsev B.A. in consultation with Petrovskiy V.V. & Taraskina N.N.
*Stellariafischeriana*	*Stellariafischeriana* (1)	Plieva T.V. & Yurtsev B.A. in consultation with Sandomirskaya S.I.
*Stellariasibirica*	*Stellariasibirica* (4)	Yurtsev B.A. in consultation with Khokhryakov A.P.
*Arenariaredowskii*, *A.tschuktschorum*	*Arenariaredowskii* (1), *Eremogonetschuktschorum* (2)	Petrovskiy V.V. & Taraskina N.N.
*Silenechamarensis*	*Silenechamarensis* (3)	Penkovskaya E.F., Siplivinskiy V.N. in consultation with Kashin L.I.
*Silenepaucifolia*	Silenechamarensisssp.paucifolia (1)	Matveeva N.V., Yurtsev B.A. in consultation with Taraskina N.N.
*Silenestenophylla*	*Silenestenophylla* (1)	Matveeva N.V., Yurtsev B.A. in consultation with Taraskina N.N. & Filipjeva E.O
*Melandtiumtriste*	*Gastrolychnistristis* (3)	Bardunov L.V. in consultation with Penkovskaya E.F.
*Gypsophilasambukii*	*Gypsophilasambukii* (1)	Plieva T.V., Yurtsev B.A. in consultation with Busik V.V., Petrochenko Yu.N. & Sandomirskaya S.I.
*Gypsophilauralensis*	*Gypsophilauralensis* (1)	Igoshina K.N. in consultation with Latsenkova A.N. & Storozhevaya M.M.
*Gypsophilaviolaceae*	*Gypsophilaviolaceae* (4)	Shreter A.I. in consultation with Khokhryakov A.P.
*Trolliusapertus*	*Trolliusapertus* (1)	Igoshina K.N. in consultation with Storozheva M.M.
*Callianthemumisopyroides*	*Callianthemumisopyroides* (3)	Vodopyanova N.S. in consultation with Malyshev L.I.
*Callianthemumsajanense*	*Callianthemumsajanense* (3)	Vodopyanova N.S. in consultation with Goloskokov V.P., Ivanova M.M., Kashina L.I., Krasnoborov I.M. & Malyshev L.I.
*Schibateranthissibirica*	*Eranthissibirica* (3)	Ivanova M.M. in consultation with Kransborov I.M.
*Aquilegiaborodinii*	*Aquilegiaborodinii* (3)	Vodopyanova N.S., Gudoshnikov S.V., Krasnoborov I.M., Penkovskaya E.F. in consultation with Kashina L.I. & Malyshev L.I.
*Aconitumdesoulavyi*	*Aconitumdesoulavyi* (4)	Gurzenkov N.N. & Gorovoy P.G.
*Aconitumpaskoi*	*Aconitumpascoi* (3)	Krasnoborov I.M. in consultation with Penkovskaya E.F.
*Aconitumsajanense*	*Aconitumsajanense* (3)	Gudoshnikov S.V.
*Anemonebiarmiensis*	*Anemonastrumbiarmiensis* (1)	Igoshina K.N. in consultation with Storozhevaya M.M. & Laschenkova A.N.
*Anemonesibirica*	*Anemonastrumsibiricum* (1)	Siplivinskiy V.N. in consultation with Kiseleva A.A., Krasnoborov I.M., Pavlov E.I. & Yurtsev B.A.
*Miyakeaintegrifolia*	*Miyakeaintegrifolia* (4)	Gorovoy P.P., Gurzenkov N.N & Egorova E.M.
*Oxygraphisglacialis*	*Oxygraphisglacialis* (1)	Kiseleva A.A. in consultation with Korobkov A.A., Petrovskiy V.V., Taraskina N.N. & Yurtsev B.A.
*Papavercanescens*	*Papavercanescens* (3)	Vodopyanova N.S., Penkovskaya E.F. in consultation with Goloskokov V.P., Ivanova M.M., Malyshev L.I. & Siplivinskiy V.N.
*Papavernivale*	*Papavernivale* (2)	Yurtsev B.A. in consultation with Khokhryakov A.P.
*Dicentraperegrina*	*Dicentraperegrina* (1)	Pimenov M.G. in consultation with Gorovoy P.P., Taraskina N.N. & Yurtsev B.A.
*Corydalisarctica*	*Corydalisarctica* (1)	Plieva T.V., Yurtsev B.A. in consultation with Sandomirskaya S.I. & Taraskina N.N.
*Corydalispauciflora*	*Corydalispauciflora* (3)	Vodopyanova N.S., Krasnoborov I.M. in consultation with Goloskokov V.P., Ivanova M.M. & Malyshev L.I.
*Macropodiumnivale*	*Macropodiumnivale* (3)	Kiseleva A.A. in consultation with Polozhiy A.V.
*Smelovskiainopinata*, *Crepisburejensis*	*Smelovskiainopinata* (4), *Crepisburejensis* (4)	Gorovoy P.G.
*Parryagrandiflora*	*Pachyneurumgrandiflorum* (3)	Gudoshnikov S.V.
*Ermaniaparryoides*	*Ermaniaparryoides* (1)	Taraskina N.N. & Yurtsev B.A.
*Gorodkoviajacutica*	*Gorodkoviajacutica* (2)	Yurtsev B.A. in consultation with Galaktionova T.V. & Taraskina N.N.
*Borodiniabaicalensis*	*Borodiniabaicalensis* (3)	Petrochenko Yu.N.
*Drabaochroleuca*	*Drabaochroleuca* (1)	Malyshev L.I. in consultation with Vodopyanova N.S., Goloskokov V.P., Mikhalyova V.M., Taraskina N.N. & Tolmachev A.I.
*Drabaturczaninovii*	*Drabaturczaninovii* (3)	Malyshev L.I. in consultation with Vodopyanova N.S. & Penkovskaya E.F.
*Rhodiolaquadrifida*	*Rhodiolaquadrifida* (1)	Bardunov L.V. in consultation with Penkovskaya E.F. & Petrochenko Yu. N.
*Bergeniacrassifolia*	*Bergeniacrassifolia* (3)	Ivanova M.M. in consultation with Krasnoborov I.M., Vodopyanova N.S., Goloskokov V.P., Kashina L.I. & Malyshev L.I.
*Bergeniapacifica*	*Bergeniapacifica* (4)	Gorovoy P.G & Gurzenkov N.N.
*Saxifragaalgisii*	*Saxifragaalgisii* (1)	Petrochenko Yu. N. & Siplivinskiy V.N.
*Saxifragaandrosacea*	*Saxifragaandrosacea* (3)	Kiseleva A.A.
*Saxifragadahurica*	*Micranthesdavurica* (1)	Petrochenko Yu. N. in consultation with Siplivinskiy V.N., Taraskina N.N., Yurtzev B.A.
*Saxifragamerkii*	*Saxifragamerkii* (1)	Siplivinskiy V.N. in consultation with Kiseleva A.A., Yurtzev B.A.
*Saxifragamelaleuca*	*Saxifragamelaleuca* (3)	Petrochenko Yu. N., Krasnoborov I.M. in consultation with Siplivinskiy V.N.
*Saxifragamultiflora*	*Saxifragaomolojensis* (2)	Yurtsev B.A. in consultation with Khokhryakov A.P., Voroshilov V.N., Petrovskiy V.V. & Taraskina N.N.
*Saxifragaredowskiana*	*Saxifragapunctata* (1)	Korobkov A.A., Yurtsev B.A. in consultation with Gorovoy P.G & Khokhryakov A.P.
*Saxifragaterektensis*	*Saxifragaterektensis* (3)	Siplivinskiy V.N. in consultation with Kiseleva A.A., Penkovskaya E.F.
*Ribesaltissimum*	*Ribesaltissimum* (3)	Vodopyanova N.S. in consultation with Goloskokov V.P., Ivanova M.M., Kashina L.I. & Malyshev L.I.
*Ribesfragrans*	*Ribesfragrans* (1)	Siplivinskiy V.N. in consultation with Kiseleva A.A., Petrochenko Yu.N., Taraskina N.N., Khokhryakov A.P. & Yurtsev B.A.
*Ribesgraveolens*	*Ribesgraveolens* (3)	Vodopyanova N.S., Gudoshnikov S.V. & Krasnoborov I.M.
*Potentillaaltaica*, *Saussureapoljakovii*	*Potentillaaltaica* (3), *Saussureapoljakowii* (3)	Vodopyanova N.S.
*Potentillabiflora*	*Potentillabiflora* (1)	Busik V.V. in consultation with Taraskina N.N. & Yurtsev B.A.
*Potentillaelegans*	*Potentillaelegans* (1)	Petrovskiy V.V., Taraskina N.N. in consultation with Krasnoborov I.M. & Malyshev L.I.
*Sieversiapentapetala*, *S.pusilla*	*Sieversiapentapetala* (4), *S.pusilla* (1)	Rebristaya O.V.
*Novosieversiaglacialis*	*Acomastylisglacialis* (1)	Rebristaya O.V. in consultation with Vodopyanova N.S., Malyshev L.I., Petrochenko Yu. N. & Taraskina N.N.
*Dryascrenulata*	*Dryascrenulata* (1)	Plieva T.V., Yurtsev B.A. in consultation with Galaktionova T.B., Sandomirskaya S.I. & Taraskina N.N.
*Dryasgrandis*	*Dryasgrandis* (1)	Plieva T.V., Yurtsev B.A. in consultation with Bylova T.V., Kiseleva A.A., Sandomirskaya S.I. & Taraskina N.N.
*Dryasoxyodonta*	*Dryasoxyodonta* (3)	Malyshev L.I. in consultation with Vodopyanova N.S., Goloskokov V.P., Kashina L.I., Krasnoborov I.M.& Polozhiy A.V.
*Dryastschonoskii*	*Dryastschonoskii* (4)	Gorovoy P.G. in consultation with Egorova E.M. & Voroshilov V.N.
*Sanguisorbaalpina*	*Sanguisorbaalpina* (3)	Kiseleva A.A. in consultation with Kashina L.I., Goloskokov V.P., Krasnoborov I.M. & Polozhiy A.V.
*Rosasichotealinensis*, *Oxytropisajanensis*	*Rosasichotealinensis* (4), *Oxytropisajanensis* (4)	Gorovoy P.G. & Gurzenkov N.N.
*Trifoliumeximium*	*Trifoliumeximium* (1)	Ivanova M.M., Krasnoborov I.M., Polozhiy A.V. in consultation with Vodopyanova N.S.
*Astragalussaralensis*	*Astragalussaralensis* (3)	Malyshev L.I. in consultation with Vodopyanova N.S., Penkovskaya E.F.
*Oxytropisaltaica*	*Oxytropisaltaica* (3)	Ivanova M.M., Polozhiy A.V. in consultation with Krasnoborov I.M.
*Oxytropisheterotricha*, *O.kodarensis*	*Oxytropisheterotricha* (3), *O.kodarensis* (3)	Malyshev L.I. & Yurtsev B.A.
*Oxytropismertensiana*	*Oxytropismertensiana* (1)	Plieva T.V. in consultation with Bylova T.V., Sandomirskaya S.I. & Yurtsev B.A.
*Oxytropisnigrescens*	*Oxytropisnigrescens* (1)	Plieva T.V., Yurtsev B.A. in consultation with Bylova T.V., Sandomirskaya S.I. & Tolmachev A.I.
*Oxytropisochotensis*	*Oxytropisochotensis* (2)	Taraskina N.N., Yurtsev B.A. in consultation with Khokhryakov A.P.
*Hedysaruminundatum*	*Hedysaruminundatum* (3)	Ivanova M.M. in consultation with Vodopyanova N.S. & Taraskina N.I.
*Linumboreale*	*Linumboreale* (1)	Igoshina K.N.
*Bupleurumeuphorbiodes*, *B.triradiatum*, *Primulacuneifolia*	*Bupleurumeuphorbiodes* (4), *B.triradiatum* (1), *Primulacuneifolia* (1)	Gorovoy P.G.
*Bupleurummartjanovii*	*Bupleurummartjanovii* (3)	Krasnoborov I.M. in consultation with Gudoshnikov S.V.
*Libanotismonstrosa*	*Sajanellamonstrosa* (3)	Gudoshnikov S.V.
*Schultziacrinita*	*Schultziacrinita* (3)	Busik V.V. in consultation with Gudoshnikov S.V. & Krasnoborov I.M.
*Tilingiaajanensis*	*Tilingiaajanensis* (1)	Belyj N.F., Gorovoy P.G., Pimenov M.G.. in consultation with Tikhomirov V.N.
*Lithosciadiummulticaule*	*Lithosciadiummulticaule* (3)	Ivanova M.M. & Siplivinskiy V.N.
*Ligusticummongolicum*	*Hanseniamongolica* (3)	Busik V.V. & Pimenov M.G.
*Conioselinumvictoris*	*Magadaniavictoris* (4)	Gorovoy P.G. & Belyj N.F.
*Angelicasaxatilis*	*Angelicasaxatilis* (1)	Pimenov M.G.
*Phlojodicarpusvillosus*	*Phlojodicarpusvillosus* (1)	Pimenov M.G. in consultation with Gorovoy P.G. & Taraskina N.N.
*Phlojodicarpuseryngiifolium*	*Kitagawiaeryngiifolia* (4)	Gorovoy P.G. in consultation with Sakhno V.G.
*Rhododendronadamsii*	*Rhododendronadamsii* (1)	Ivanova M.M., Krasnoborov I.M. in consultation with Galaktionova T.F., Mikhalyova V.M. & Plieva T.V.
*Rhododendronaureum*	*Rhododendronaureum* (1)	Krasnoborov I.M. in consultation with Busik V.V., Gorovoy P.G., Kashina L.I., Mikhalyova V.M. & Taraskina N.N.
*Rhododendronredowskianum*	*Rhododendronredowskianum* (1)	Siplivinskiy V.N. in consultation with Busik V.V., Mikhalyova V.M., Taraskina N.N. & Yurtsev B.A.
*Phyllodocealeutica*	*Phyllodocealeutica* (4)	Gorovoy P.G. in consultation with Voroshilova V.N. & Egorova E.M.
*Bryanthusgmelinii*, *Arctericanana*, *Campanulachamissonis*, *Popoviocodoniastenocarpa*, *P.uyemurae*	*Bryanthusgmelinii* (4), *Arctericanana* (4), *Campanulachamissonis* (4), *Popoviocodoniastenocarpa* (4), *Campanulauyemurae* (4)	Shreter A.I.
*Cassiopelycopodiodes*	*Cassiopelycopodiodes* (4)	Pimenov M.G. in consultation with Gorovoy P.G.
*Cassioperedowskii*	*Cassioperedowskii* (4)	Pimenov M.G.
*Diapensiaobovata*	*Diapensiaobovata* (1)	Siplivinskiy V.N. in consultation with Busik V.V., Mikhalyova B.M. & Taraskina N.N.
*Androsaceochotensis*	*Androsaceochotensis* (2)	Petrovskiy V.V. & Taraskina N.N.
*Gentianafalcata*	*Comastomafalcatum* (3)	Vodopyanova N.S. in consultation with Goloskokov V.P.
*Gentianagrandiflora*	*Ciminalisgrandiflora* (3)	Bardunov L.V. in consultation with Krasnoborov I.M. & Petrochenko Yu.N.
*Gentianauniflora*	*Calathianauniflora* (1)	Vodopyanova N.S. in consultation with Goloskokov V.P., Kashina L.I. & Malyshev L.I.
*Swertiakomarovii*	*Swertiakomarovii* (3)	Ivanova M.M. in consultation with Vodopyanova N.S.
*Polemoniumpulchellum*, *Mertensiastylosa*	*Polemoniumpulchellum* (3), *Mertensiastylosa* (3)	Ivanova M.M. in consultation with Krasnoborov I.M.
*Mertensiarivularis*	*Mertensiarivularis* (4)	Shreter A.I. in consultation with Krasnoborov I.M.
*Dracocephalumpopovii*	*Dracocephalumpopovii* (3)	Busik V.V. & Siplivinskiy V.N.
*Phlomiskoraiensis*	*Phlomiskoraiensis* (4)	Gorovoy P.G & Gurzenkov N.N.
*Veronicasajanensis*	*Veronicasajanensis* (3)	Krasnoborov I.M. in consultation with Kashina L.I.
*Pedicularisadamsii*	*Pedicularisalopecuroides* (1)	Vodopyanova N.S. in consultation with Malyshev L.I., Mikhalyova V.M., Taraskina N.N. & Yurtsev B.A.
*Pedicularisamoena*	*Pedicularisamoena* (1)	Plieva T.V., Yurtsev B.A. in consultation with Busik V.V., Ivanina L.I. & Sandomirskaya S.I.
*Pedicularisarguserrata*	*Pedicularisanthemifolia* (3)	Krasnoborov I.M. in consultation with Kashina L.I. & Malyshev L.I.
*Pedicularisbrachystachys*	*Pedicularisbrachystachys* (3)	Gudoshnikov S.V. in consultation with Malyshev L.I.
*Pediculariscompacta*	*Pediculariscompacta* (1)	Vodopyanova N.S. in consultation with Goloskokov V.P., Ivanova M.M., Kashina L.I., Krasnoborov I.M., Malyshev L.I. & Storozhevaya M.M.
*Pediculariseriophora*	*Pediculariseriophora* (4)	Plieva T.V. & Yurtsev B.A.
*Pedicularisfissa*	*Pedicularisfissa* (3)	Vodopyanova N.S. in consultation with Ivanova M.M., Kashina L.I., Krasnoborov I.M. & Malyshev L.I.
*Pedicularistristis*	*Pedicularistristis* (1)	Vodopyanova N.S. in consultation with Goloskokov V.P., Gorovoy P.G., Ivanova M.M., Kashina L.I., Malyshev L.I., Mikhalyova V.M., Penkovskaya E.F., Plieva T.V. & Yurtsev B.A.
*Valerianaturczaninowii*	*Valerianaaltaica* (3)	Ivanova M.M., Penkovskaya E.F., Siplivinskiy V.N. in consultation with Kashina L.I.
*Campanuladasyantha*	*Campanuladasyantha* (1)	Vodopyanova N.S. in consultation with Galaktionova G.F., Gorovoy P.G., Ivanova M.M., Kashina L.I., Krasnoborov I.M. & Malyshev L.I.
*Astrocodonexpansus*	*Astrocodonexpansus* (4)	Shreter A.I. in consultation with Gorovoy P.G., Taraskina N.N. & Yurtsev B.A.
*Erigeronflaccidus*	*Erigeronflaccidus* (1)	Vodopyanova N.S. in consultation with Ivanova M.M., Malyshev L.I., Penkovskaya E.F. & Petrochenko Yu.N.
*Pyrethrumpulchellum*	*Pyrethrumpulchellum* (3)	Vodopyanova N.S., Gudoshnikov S.V. in consultation with Ivanova M.M., Kashina L.I. & Krasnoborov I.M.
*Artemisiaflava*	*Artemisiaflava* (2)	Korobkov A.A.
*Artemisiafurcata*	*Artemisiafurcata* (1)	Plieva T.V., Yurtsev B.A. in consultation with Galaktionova G.F., Korobkov A.A. & Sandomirskaya S.I.
*Artemisiaglomerata*	*Artemisiaglomerata* (1)	Pimenov M.G.
*Artemisiakruhsiana*, *A.senjavinensis*	*Artemisiakruhsiana* (2), *A.senjavinensis* (2)	Korobkov A.A.
*Artemisialagopus*	*Artemisialagopus* (1)	Plieva T.V., Yurtsev B.A. in consultation with Galaktionova G.F., Korobkov A.A. & Sandomirskaya S.I.
*Nardosmiaglacialis*, *N.gmelinii*	*Petasitesglacialis* (2), *P.sibiricus* (2)	Petrovskiy V.V. & Taraskina N.N.
*Nardosmiasaxatilis*	*Petasitesrubellus* (1)	Krasnoborov I.M. in consultation with Kashina L.I. & Kiseleva A.A.
*Seneciojacuticus*	*Tephroserisjacutica* (1)	Yurtsev B.A. in consultation with Gorovoy P.G, Kiseleva A.A., Malyshev L.I., Mikhalyova V.M. & Khokhryakov A.P.
*Aconitumsichotense*, *Seneciosichotensis*, *Lugulariasichotensis*, *Saussureaajanensis*, *S.porcellanea*, *S.sovietica*, *Hieraciumcoreanum*	*Aconitumsichotense* (4), *Tephroserissichotensis* (4), *Lugulariasichotensis* (4), *Saussureaajanensis* (4), *S.porcellanea* (4), *S.sovietica* (4), *Hieraciumcoreanum* (4)	Gurzenkov N.N. & Gorovoy P.G.
*Seneciotuczaninovii*	*Tephroseristurczaninovii* (3)	Vodopyanova N.S. in consultation with Ivanova M.M., Krasnoborov I.M. & Malyshev L.I.
*Saussureabaicalensis*	*Saussureabaicalensis* (3)	Vodopyanova N.S., Ivanova M.M. & Malyshev L.I.
*Saussureacongesta*	*Saussureacongesta* (3)	Vodopyanova N.S. in consultation with Ivanova M.M., Kashina L.I., Krasnoborov I.M. & Malyshev L.I.
*Saussureafrolovii*, *S.sajanensis*, *Taraxacumaltaicum*	*Saussureafrolovii* (3), *S.sajanensis* (3), *Taraxacumaltaicum* (3)	Gudoshnikov S.V.
*Saussurealatifolia*	*Saussurealatifolia* (3)	Gudoshnikov S.V., Vodopyanova N.S., Krasnoborov I.M. in consultation with Goloskokov V.P., Ivanova M.M. & Kashina L.I.
*Saussureaschanginiana*	*Saussureaschanginiana* (1)	Vodopyanova N.S. in consultation with Goloskokov V.P., Ivanova M.M., Kashina L.I., Krasnoborov I.M., Malyshev L.I., Mikhalyova V.M. & Yurtsev B.A.
*Saussureauralensis*	*Saussureauralensis* (1)	Igoshina K.N. in consultation with Storozhevaya M.M.
*Taraxacumglabrum*	*Taraxacumglabrum* (3)	Malyshev L.I. in consultation with Vodopyanova N.S., Goloskokov V.P. & Krasnoborov I.M.
*Crepischrysantha*	*Crepischrysantha* (1)	Krasnoborov I.M. in consultation with Kashina L.I., Kiseleva A.A.
*Crepisgmelinii*	*Crepisgmelinii* (1)	Yurtsev B.A. in consultation with Galaktionova T.V.

**Table 2.** Taxonomic distribution of endemic alpine species of northern Asia in the dataset. Families are listed in descending order of the number of species.

№	Family	Num. of species	Num. of records
1	Asteraceae Bercht. & J. Presl	37	2054
2	Fabaceae Lindl.	17	484
3	Poaceae Barnhart	16	579
4	Saxifragaceae Juss.	16	860
5	Brassicaceae Burnett	14	396
6	Salicaceae Mirb.	14	978
7	Ranunculaceae Juss.	13	725
8	Rosaceae Juss.	12	854
9	Apiaceae Lindl.	11	958
10	Caryophyllaceae Juss.	11	895
11	Scrophulariaceae Juss.	9	721
12	Ericaceae Juss.	8	735
13	Cyperaceae Juss.	6	424
14	Papaveraceae	6	387
15	Campanulaceae Juss.	5	200
16	Gentianaceae Juss.	5	298
17	Portulacaceae Juss.	5	317
18	Primulaceae Batsch ex Borkh.	4	137
19	Grossulariaceae D.C.	3	249
20	Lamiaceae Martinov	3	39
21	Betulaceae Gray	2	595
22	Boraginaceae Juss.	2	49
23	Crassulaceae J.St.-Hil.	2	131
24	Cupressaceae Gray	2	206
25	Lentibulariaceae Rich.	2	47
26	Pteridaceae E.D. M. Kirchn.	1	28
27	Diapensiaceae Lindl.	1	213
28	Juncaceae Juss.	1	59
29	Linaceae DC. ex Perleb	1	22
30	Polemoniaceae Juss.	1	27
31	Valerianaceae Batsch	1	42

**Table 3.** Species and its number of distribution records in the dataset. Species records are listed in descending order.

*Betulamiddendorffii*	415	*Artemisiaglomerata*	103	*Paracolpodiumaltaicum*	57
*Rhododendronaureum*	401	*Rhodiolaquadrifida*	103	*Salixsajanensis*	56
*Schulziacrinita*	306	*Ribesaltissimum*	103	*Tephroserisjacutica*	56
*Anemonastrumsibiricum*	290	*Salixtschuktschorum*	103	*Salixtorulosa*	55
*Silenechamarensis*	276	*Dryasgrandis*	102	*Taraxacumglabrum*	55
*Crepischrysantha*	239	*Corydalisarctica*	101	*Saxifragaterektensis*	54
*Pedicularisamoena*	220	*Oxytropisnigrescens*	96	*Poapseudoabbreviata*	53
*Diapensiaobovata*	213	*Hedysaruminundatum*	93	*Crepispolytricha*	52
*Carexledebouriana*	209	Silenechamarensisssp.paucifolia	91	*Callianthemumisopyroides*	51
*Bergeniacrassifolia*	204	*Corydalispauciflora*	90	*Oxytropisaltaica*	50
*Salixsphenophylla*	191	*Dicentraperegrina*	90	*Saxifragaandrosacea*	50
*Betularotundifolia*	180	*Leymusinterior*	90	*Trifoliumeximium*	49
*Juniperuspseudosabina*	179	*Claytoniajoanneana*	88	*Saxifragaalgizii*	49
*Acomastylisglacialis*	171	*Petasitesglacialis*	86	*Primulacuneifolia*	47
*Pediculariscompacta*	164	*Poaaltaica*	86	*Salixfimbriata*	47
*Potentillaelegans*	161	*Oxygraphisglacialis*	85	*Drabaochroleuca*	46
*Tilingiaajanensis*	160	*Papavercanescens*	85	*Salixnasarovii*	46
*Petasitessibiricus*	139	*Stellariafischeriana*	85	*Ermaniaparryoides*	45
*Artemisiafurcata*	136	*Saussureacongesta*	83	*Gorodkoviajacutica*	44
*Ciminalisgrandiflora*	135	*Gypsophilauralensis*	81	*Hyalopoalanatiflora*	43
*Claytoniaacutifolia*	135	*Artemisiakruhsiana*	77	*Pedicularisanthemifolia*	43
*Forniciumcarthamoides*	135	*Gastrolychnistristis*	76	*Pedicularisfissa*	43
*Macropodiumnivale*	132	*Saxifragamerkii*	75	*Valerianaaltaica*	42
*Bupleurumtriradiatum*	130	*Hanseniamongolica*	74	*Sieversiapentapetala*	41
*Rhododendronadamsii*	129	*Tephroseristurczaninovii*	74	*Sieversiapusilla*	41
*Salixrectijulis*	125	*Eremogonetschuktschorum*	73	*Pedicularisbrachystachys*	40
*Salixturczaninowii*	125	*Saxifragapunctata*	73	*Lithosciadiummulticaule*	39
*Phlojodicarpusvillosus*	124	*Rhododendronredowskianum*	72	*Taraxacumaltaicum*	39
*Campanuladasyantha*	121	*Sajanellamonstrosa*	72	*Aquilegiaborodinii*	37
*Eriophorumhumile*	117	*Pyrethrumpulchellum*	70	*Helictotrichonmongolicum*	37
*Sanguisorbaalpina*	116	*Angelicasaxatilis*	69	*Oxytropiskusnetzovii*	37
*Saussureaschanginiana*	115	*Callianthemumsajanense*	65	*Ribesgraveolens*	36
*Salixdivaricata*	114	*Potentillabiflora*	65	*Salixbrayi*	36
*Saussurealatifolia*	114	*Saxifragaredofskyi*	65	*Saussureafrolowii*	36
*Pedicularistristis*	113	*Androsaceochotensis*	64	*Trolliusapertus*	36
*Silenestenophylla*	112	*Artemisialagopus*	63	*Drabaturczaninowii*	35
*Anemonastrumbiarmiensis*	111	*Scirpusmaximowiczii*	61	*Aconitumpascoi*	33
*Calanthianauniflora*	110	*Claytoniaarctica*	60	*Saussureabaicalensis*	33
*Dryasoxyodonta*	110	*Salixberberifolia*	60	*Saussureapoljakowii*	32
*Ribesfragrans*	110	*Gypsophilasambukii*	59	*Saxifragadavurica*	32
*Saxifragamelaleuca*	110	*Luzulaarcuata*	59	*Pinguiculaspathulata*	31
*Petasitesrubellus*	105	*Cassiopelycopodioides*	58	*Bryanthusgmelinii*	30
*Erigeronflaccidus*	104	*Pedicularisalopecuroides*	58	*Claytoniaeschscholitzii*	30
*Dracocephalumfragile*	30	*Chrysospleniumpeltatum*	17	*Oxytropisheterotricha*	7
*Mertensiastylosa*	30	*Drabapygmaea*	17	*Aconitumdesoulavyi*	6
*Ptilagrostisjunatovii*	30	*Kitagawiaeryngiifolia*	17	*Magadaniavictoris*	6
*Oxytropisochotensis*	29	*Saxifragakruhsiana*	17	*Swertiabaicalensis*	6
*Saxifragabrachypetala*	29	*Festucachionobia*	16	*Taraxacumsoczavae*	6
*Campanulachamissonis*	28	*Kreczetovicziauniflora*	16	*Cardaminepedata*	5
*Cryptogrammaraddeana*	28	*Pinguiculaalgida*	16	*Dracocephalumpopovii*	5
*Eranthissibirica*	28	*Poaircutica*	16	*Salixkhokhrjakovii*	5
*Rhodiolapinnatifida*	28	*Chrysospleniumalberti*	15	*Poaivanoviae*	4
*Crepisgmelinii*	27	*Koeleriageniculata*	15	*Phlomiskoraiensis*	4
*Microbiotadecussata*	27	*Pediculariseriophora*	15	*Cardamineconferta*	4
*Polemoniumpulchellum*	27	*Potentillaaltaica*	15	*Claytoniavassilievii*	4
*Saxifragamultiflora*	27	*Phyllodocealeutica*	14	*Crepisburejensis*	4
*Bupleurummatrjanovii*	26	*Salixkamtschatica*	13	*Miyakeaintegrifolia*	4
*Comastomafalcatum*	26	*Borodiniabaicalensis*	12	Oxytropisajanensisssp.semiglobosa	4
*Astragalussaralensis*	25	*Arctericanana*	12	*Oxytropiskodarensis*	4
*Astrocodonexpansus*	25	*Arenariaredowskii*	12	*Oxytropisoxyphylloides*	4
*Veronicasajanensis*	25	*Cardaminevictoris*	12	*Oxytropissumneviczii*	3
*Androsacegorodkovii*	24	*Oxytropissajanensis*	12	*Aconitumsichotense*	3
*Chrysospleniumbaicalense*	23	*Saussureauralensis*	12	*Artemisiaflava*	3
*Gypsophilaviolacea*	23	*Arabisturczaninowii*	11	*Delphiniumsajanense*	3
*Helictotrichonkrylovii*	23	*Corydalisgorodkovii*	11	*Ligulariasichotensis*	3
*Oxytropismertensiana*	23	*Oxytropisjurtzevii*	11	*Saussureaporcellanea*	3
*Pachyneurumgrandiflorum*	23	*Saussureasquarrosa*	11	*Tephroserissichotensis*	3
*Linumboreale*	22	*Artemisiasenjavinensis*	10	*Rosa sichotealinensis*	2
*Swertiakomarovii*	21	*Papavernivale*	10	*Androsacesemiperennis*	2
*Dryascrenulata*	21	*Dryastschonoskii*	9	*Hieraciumcoreanum*	2
*Bergeniapacifica*	20	*Aconitumsajanense*	9	*Salixjurtzevii*	2
*Carexalticola*	20	*Bupleurumeuphorbioides*	9	*Saussureasovietica*	2
*Pyrethrumlanuginosum*	20	*Koeleriaatroviolacea*	9	*Smelowskiainopinata*	2
*Cassioperedowskii*	19	*Elymussajanensis*	8	*Carexcaucasica*	1
*Mertensiarivularis*	19	*Eutremaedwardsii*	8	*Oxytropisajanensis*	1
*Popoviocodoniastenocarpa*	19	*Stellariasibirica*	7	*Saussureaajanensis*	1
*Festucasichotensis*	18	*Campanulauyemurae*	7	*Saussureasajanensis*	1
Total number of records: 13709					
